# Evaluating the Evidence Surrounding Pontine Cholinergic Involvement in REM Sleep Generation

**DOI:** 10.3389/fneur.2015.00190

**Published:** 2015-09-01

**Authors:** Kevin P. Grace, Richard L. Horner

**Affiliations:** ^1^Department of Medicine, University of Toronto, Toronto, ON, Canada; ^2^Department of Physiology, University of Toronto, Toronto, ON, Canada

**Keywords:** acetylcholine, REM sleep, pons, gain-of-function, loss-of-function, sleep dynamics, cholinomimetic

## Abstract

Rapid eye movement (REM) sleep – characterized by vivid dreaming, motor paralysis, and heightened neural activity – is one of the fundamental states of the mammalian central nervous system. Initial theories of REM sleep generation posited that induction of the state required activation of the “pontine REM sleep generator” by cholinergic inputs. Here, we review and evaluate the evidence surrounding cholinergic involvement in REM sleep generation. We submit that: (i) the capacity of pontine cholinergic neurotransmission to generate REM sleep has been firmly established by gain-of-function experiments, (ii) the function of endogenous cholinergic input to REM sleep generating sites cannot be determined by gain-of-function experiments; rather, loss-of-function studies are required, (iii) loss-of-function studies show that endogenous cholinergic input to the PTF is not required for REM sleep generation, and (iv) cholinergic input to the pontine REM sleep generating sites serve an accessory role in REM sleep generation: reinforcing non-REM-to-REM sleep transitions making them quicker and less likely to fail.

## Background

### Characterizing REM sleep as a unique state of the central nervous system

Rapid eye movement (REM) sleep – characterized by vivid dreaming, electroencephalographic activation, and motor atonia – is a fundamental operating mode of the mammalian central nervous system. Before REM sleep was discovered, sleep was assumed to be a homogenous brain state (1). However, electrophysiological recording of activity in the sleeping brain revealed sleep’s dual nature (2, 3). Pioneering electroencephalography (EEG) studies showed that cortical activity in sleeping subjects is dominated by high amplitude, low frequency oscillations; however, beginning in the 1930s, several groups reported that, during sleep, such slow-wave activity is periodically interrupted by periods of low-amplitude fast-waves resembling waking EEG (3–6). The seminal characterization of this novel sleep stage and its physiological correlates were made by Aserinsky, Kleitman, and Dement in the 1950s. They observed that periodic EEG activations during sleep occurred together with rapid, jerky, and binocularly symmetrical eye movements, which differed from the slow, rolling, and pendular eye movements that had been previously described in sleeping subjects. The authors also observed other phenomena typical of the state, including surges in respiratory rate, heightened arousal threshold, and the occurrence of “fine movements” in the extremities (i.e., muscle twitching). Perhaps their most intriguing observation was the association between this collection of phenomena and dreaming. Subjects awoken from periods of REM reported an increased incidence of highly visual and detailed dreams.

The discovery of the REM stage of sleep presented a conceptual challenge to the field of sleep research. Whether this stage was a *distinct* sleep state, or whether all sleep stages stemmed from a common neural mechanism was still uncertain. The distinction between these two perspectives is non-trivial. REM sleep being a distinct state would mean that distinct neural circuitry is responsible for its generation, circuitry giving rise to unique physiology, pathophysiology, and functionality. Aserinsky, Kleitman, and Dement described the REM and non-REM sleep stages, not as distinct states but, as different “depths” of the same biological phenomenon. It was suggested that sleep cycling is a process by which the brain progresses through deeper and deeper stages of sleep followed by a “lightening” associated with the occurrence of EEG activation with REMs (3). The REM sleep stage was characterized as a “manifestation of a particular level of cortical activity which is encountered normally during sleep” (2); that level of cortical activity being one that is intermediate between wakefulness and slow-wave sleep. Aserinsky, Kleitman, and Dement characterized the activated EEG associated with REMs as being equivalent to the electroencephalographic signature of Stage 1 sleep, which corresponded to the “drowsy” stage that had been previously described by Gibbs and Gibbs (7). Speaking of the Stage 1 sleep that occurs at sleep onset, Dement and Kleitman commented that “this Stage 1 EEG seemed to be identical with those occurring later in the night concomitant with actual eye movements” (3).

The suggestion that REM sleep represents one of the lightest stages of sleep conflicted with data showing that arousal threshold is highest during periods of REM and EEG activation (3). Building on the descriptive work of Aserinsky, Kleitman, and Dement, a series of experiments and philosophical arguments (1958–1960) authored by Michel Jouvet is largely responsible for the current conception of REM sleep as a brain state distinct from both non-REM sleep and wakefulness (8, 9). Jouvet reported that the occurrence of rapid cortical activity together with the so-called “somato-vegetative phenomena” (i.e., the disappearance of all muscle activity as well as variation in cardiac and respiratory rhythms) persisted following various supra-pontine transections in cats, but were eliminated following sectioning between the pons and trapezoid bodies (8). Subsequent lesion experiments showed that destroying portions of the pontine tegmental field (PTF) (specifically the caudal pontine reticular formation and the posterior part of the oral pontine reticular formation) caused a selective loss of the REM sleep stage (8). Moreover, the REM sleep stage could be selectively triggered by timed electrical stimulation of this same region. These findings were difficult to reconcile with the perspective of Kleitman and colleagues that REM sleep is an intermediary stage between wakefulness and non-REM sleep produced by the combined action of wake and slow-wave sleep circuitry. These data indicated that the REM sleep stage was mechanistically unique because it depended upon the integrity of pontine control circuitry that was not involved in the generation of other sleep stages. Having identified a neuroanatomical locus for the REM sleep stage, Jouvet was able to argue that the phenomenological components of the REM sleep stage constituted a distinct state of the central nervous system (8, 10–12).

## A Cholinergic Mechanism of REM Sleep Generation

### Early developments

The recognition of REM sleep as an independent sleep state led to speculation regarding the possible mechanisms responsible for its generation. At the CIBA Foundation symposium on the nature of sleep in 1960, Michel Jouvet reported that the induction of REM sleep by electrical stimulation of the PTF was followed by a “refractory” period during which identical stimulations resulted in wakefulness accompanied by hypertonia and agitation. He remarked that the existence of this refractory period was suggestive of a “neurohumoral mechanism [of REM sleep generation] which would “discharge” periodically during behavioural sleep but which could not be brought into play until a sufficient “stock” of neurohormones was gathered.” He further speculated that this neurohumoral mechanism may importantly involve cholinergic neurotransmission (8). Stemming from Jouvet’s initial hypothesis, numerous studies have investigated the potential involvement of cholinergic neurotransmission in REM sleep generation; however, there is presently no consensus viewpoint. This main focus of this review will be to evaluate the evidence relating to the potential involvement of cholinergic neurotransmission in REM sleep generation. It is our position that a consensus viewpoint can be reached because the bulk of available evidence can be brought into agreement when appropriately interpreted and considered in light of recent findings.

Speculation over cholinergic involvement in the mechanism of REM sleep generation stemmed from studies showing the REM sleep modulating effects of systemically administered cholinergic drugs (10, 11). In cats, systemic administration of the cholinergic receptor antagonist atropine strongly disrupts sleep and wakefulness. Systemically administered atropine results in what was described as a “dissociation between the behaviour of the animal and its electrical activity” (11). While the EEG of atropinized cats was marked by slow-waves and bursting activity similar to that observed during natural sleep and anesthesia, they were nevertheless awake. These animals had tonically elevated muscle activity, adopted standing, and crouching postures, and remained responsive to auditory stimulation. However, auditory or noxious stimulation did not elicit cortical activation. Atropinized cats did not have REM sleep. Nevertheless, consistent with there being some *capacity* of cholinergic neurotransmission to promote REM sleep generation, increasing endogenous levels of acetylcholine, by systemic administration of an acetylcholinesterase inhibitor, increased REM sleep bout durations as well as the intensity of phasic REM phenomena (10, 11).

### Cholinergic stimulation of the putative REM sleep generator in the pontine tegmental field

#### Features of REM Sleep Induction by Cholinergic Agonism in the Pontine Tegmental Field

##### Overview of seminal findings

The reported link between cholinergic neurotransmission and REM sleep generation gave rise to the hypothesis that cholinergic activation of the putative REM sleep generating circuitry in the PTF is the critical event leading to the initiation of the state. To-date, the bulk of evidence cited in support of this hypothesis comes from studies showing that stimulating sites in the pons with cholinergic agonists is sufficient to induce REM sleep. The seminal cholinergic stimulation study by George et al. (13) showed that microinjections of cholinomimetics (i.e., carbachol and oxotremorine) in cats, at PTF sites where lesioning eliminated REM sleep, produced extended REM sleep-like states. These states were characterized by atonia, electrocortical activation, reflex inactivation, and REMs. Induced REM sleep persisted for 45–50 min and occurred at a short latency following microinjection (1–5 min). The latency to and magnitude of the induced REM sleep are important details because they suggest that the PTF REM sleep circuitry is structured such that cholinergic inputs are capable of overwhelming competing influences. This “capacity to overwhelm” was the primary indicator that cholinergic neurotransmission might play a major role in REM sleep generation. Cholinergic PTF stimulation also induced dissociated states where motor atonia or a REM sleep-like EEG theta rhythm occurred apart from other REM sleep phenomena. The finding that dissociated states could be triggered by acetylcholine in addition to fully orchestrated REM sleep is significant for three reasons. First, the induction of dissociated states suggests that the PTF is a source of multiple efferent pathways that can independently control components of the REM sleep state. Second, the occurrence of fully orchestrated REM sleep (i.e., marked by the synchronized onset and offset of the full complement of REM sleep components) indicates that the PTF is also a likely source of controller/switching circuitry – responsible for coordinating the activation of REM sleep components, while preventing the intrusion of other behavioral states. Finally, these findings suggest that the phenomenological afferents and the executive controller are cholinoceptive.

REM sleep induction by cholinergic PTF stimulation has been repeated by numerous other studies in both cats and rats (14–36). The experimental details of these studies are listed in Table 1. REM sleep can be elicited by microinjection of the mixed acetylcholine receptor agonist carbachol, selective muscarinic acetylcholine receptor agonists (e.g., oxotremorine and bethanechol), and acetylcholinesterase inhibitors (e.g., neostigmine). REM sleep effects exhibit dose dependency and can be blocked by co-application of cholinergic receptor antagonists (e.g., atropine). REM sleep-like states have also been induced in decerebrate and anesthetized animals [for a review of REM sleep induction in reduced preparations refer to the review by Kubin (37)].

**Table 1:** **The effects of modulating pontine cholinergic neurotransmission on REM sleep**.

Experiment type	Reference	Drug delivery	Drug	Species	PTF region	Sleep effects	Motor effects	Latency
Local gain-of-function	Cordeau et al. (14)	Microinjection	Acetylcholine (20 μg)	Cat	mPnC	Injections in awake cats: induced NREM sleep and sleep attacks; injections in sleeping cats: no effect other than further EEG slowing; REM sleep (“desynchronized” EEG pattern, no movement, twitching) induced in 4 cats		1–5 min
	George et al. (13)	Microinjection	Carbachol (0.2–5 μg); oxotremorine (0.2–10 μg)	Cat	PnO/C	Long bouts (45–50 min) of a REM sleep-like state (atonia, lost reflexes, “low voltage fast” EEG pattern, “hyper-synchronous” hippocampal activity); Mixed states: (i) atonia only, (ii) escalating ⊖ rhythm + sensory responsiveness	As the REM sleep-state waned a severe tremor emerged	1–5 min
	Kostowski (15)	Microinjection	ACh (5–15 μg); nicotine (5 μg); eserine (10 μg)	Cat	mPnO/C	Eserine and Ach: induced signs of sedation and sleep; Nicotine: biphasic effect, excitation followed by sedation	Nicotine: stiffening of the tail + torsion of the head	3–6 min
	Mitler and Dement (38)	Microinjection	Carbachol (5 μg/1 μl)	Cat	Peri-LCα/dPNO	Induced wakefulness and arousal	Motor inhibition followed arousal (40%) flaccidity and areflexia lasting 20+ h	<10 min
	Amatruda et al. (16)	Microinjection	Carbachol (3–9 μg)	Cat	PnO/C	Persistent atonia with EEG desynchronization; REM sleep time ↑3.5–4.5 times (dose dependent)		22.3 ± 25.9 min
	van Dongen et al. (17)	Microinjection	Carbachol (0.05–0.5 μg/0.5 μl) physostigmine (2–20 μg/0.5 μl)	Cat	Peri-LCα/dPNO	Carbachol: episodes of motor atonia lasting 7–19 min following 60% of injections; effects blocked by atropine but not by mecamylamine; cats appeared awake at all times; Physostigmine: no effects	Asymmetric body flexion, circling, aggression	<1–32 min
	van Dongen (19)	Microinjection	Carbachol (0.05–0.5 μg/0.5 μl)	Cat	Peri-LCα/dPNO	Motor atonia following 30% of injections; EEG not recorded	Asymmetric body flexion with/without circling (30%), aggression (9%)	<15 min
	Silberman et al. (18)	Microinjection	Carbachol (4 μg/0.25–1 μl)	Cat	PnO/C	REM sleep time ↑2–12×; length ↑2–21×		Highly variable
	Hobson et al. (20)	Microinjection	Bethanechol (1.4–7 μg)	Cat	vPnC	Long bouts (40–50 min) of a REM sleep-like state (atonia, PGO waves, unresponsive, desynchronized EEG, reversible); REM sleep time ↑3–5 times (dose dependent); Mixed states: induced at lowest dose (1.4 μg), details not specified		>25 min
	Baghdoyan et al. (21)	Microinjection	Neostigmine (0.2–20 μg/0.25 μl)	Cat	mPnO/C	REM sleep time ↑9× (bout length and frequency ↑); NREM sleep time ↓85%; effects were dose dependent and were blocked by atropine		18.5 ± 6.6 min
	Baghdoyan et al. (22)	Microinjection	Carbachol (4 μg/0.5 μl)	Cat	PnO/C	REM sleep time ↑4× (bout length and frequency ↑); NREM sleep time ↓85%		~45 min
	Gnadt and Pegram (23)	Microinjection	Carbachol (0.1–5 μg/0.1 μl)	Rat	PnO/C SubC	REM sleep ↑1.52× only with caudal pontine injections of 0.5 and 1.0 μg doses; wake ↑1.5× at the 5.0 μg dose while REM and NREM sleep were reduced	Asymmetric body flexion, ↑muscle tone, circling	39 min
	Shiromani (39)	Microinjection	Carbachol (8 μg/1 μl)	Cat	PnO/C	Long bouts (11–47 min) of a REM sleep-like state (atonia, PGO waves, desynchronized EEG pattern); Mixed states: (i) atonia only (lasting ~ 50 min), (ii) escalating ⊖ rhythm + sensory responsiveness		6.1 ± 1.8 min
	Baghdoyan et al. (26)	Microinjection	ACh (5 μg); carbachol (4 μg) (0.25–0.5 μl)	Cat	PnO/C	REM sleep time ↑3× (bout frequency ↑) (latencies ↓ and REM sleep time ↑ for rostrodorsal relative to ventrocaudal injections)		42 ± 33 min
	Vanni-Mercier et al. (27)	Microinjection	Carbachol (0.4 μg/0.2 μl)	Cat	Peri-LCα, PnO/C	Site-specific REM sleep effects (REM sleep enhancement or suppression, or no effect); peri-LCα injections were most effective: REM sleep time ↑3× (↑bout length (>20 min) or ↑frequency); NREM sleep time ↓ independent of site; Mixed states: (i) atonia, REMs, PGO waves, hippocampal ⊖ rhythm + sensory responsiveness (ii) atonia and hippocampal ⊖ rhythm only with sensory responsiveness (iii) hippocampal ⊖ rhythm persisting into in NREM sleep		Peri-LCα: 5.5 ± 0.9 min
	Velazquez-Moctezuma et al. (28)	Microinjection	Carbachol (3.6 μg/0.1 μl); McN-A-343 (M1 agonist; 1.6 μg); oxotremorine (M2 agonist; 1.6 μg)	Cat	unknown	Oxotremorine and carbachol: REM sleep time ↑3 and 4×, respectively (bout length and frequency effects were not reported); McN-A-343: no response		Carb:18.1 ± 6.4 min Oxo: NSD McN: NSD
	Yamamoto et al. (29)	Microinjection	Carbachol (4 μg/0.25 μl)	Cat	Peri-LCα, PnO	REM sleep time ↑0.5× on average; effect latency and magnitude positively and negatively correlated, respectively, with the distance of injection sites from an oblique line running anterodorsally to posteroventrally		1–40 min
	Reinoso-Suarez et al. (32)	Microinjection	Carbachol (0.8–16 μg/0.02–0.03 μl)	Cat	PnO	Dorsal sites: persistent wakefulness with periodic muscle atonia; REM sleep time ↑2.5–3 h × 4 h after injection; 5–40 min REM sleep episodes; Mixed states: atonia only; Ventral sites: REM sleep time ↑6×; Mixed states: PGO activity with muscle tone and activated EEG		Dorsal: 2.2 ± 1 min; ventral: 4.7 ± 2.2 min
	Lopez-Rodriguez et al. (40)	Iontophoretic microinjection	ACh (2M); neostigmine (2M) 200–500 nA current	Cat	Peri-LCα, PnO	Induced multiple states at identical sites depending on initial conditions. Injections during NREM = REM sleep (39% of cases), Wake (17%), N-Dis (12%), W-Dis (11%), no effect (22%); Injections during Wake = REM sleep (17% of cases), NREM (5%), N-Dis (17%), W-Dis (41%), no effect (20%); Mixed states: desynchronized EEG with atonia (W-Dis); synchronized EEG with PGO waves and muscle atonia (N-Dis)		4–8 min
	Imeri et al. (30)	Microinjection	Carbachol (0.5 μg/0.1 μl)	Rat	vPnC	REM sleep time ↑1.5×; Wake and NREM sleep were reportedly unaffected local *loss*-of-function		NA
	Mastrangelo et al. (31)	Microinjection	Carbachol (1 μg/0.5 μl)	Rat	unknown	No response in 25% of rats tested; Carb induced 20–80 min of wakefulness following injection; REM sleep ↑1.4× thereafter (bout frequency ↑1.3×)	Circling	60 min
	Bourgin et al. (33)	Microinjection	Carbachol (1–500 ng/50 nl)	Rat	PnO/C SubC	REM sleep time ↑2× (bout frequency ↑2×); effects were dose dependent and were blocked by atropine; highest doses induced wakefulness		NSD
	Deurveilher et al. (34)	Microinjection	Carbachol (0.005–3 μg/0.1 μl)	Rat	PnO/C SubC	No response (74%); carb ↑ wakefulness (2×) (13%); often associated with motor disturbances; REM sleep ↑1.5–2× (13%)	Asymmetric body flexion (8%), circling (3%), hypoactivity (1%)	NSD
	Marks and Birabil (36)	Microinjection	Carbachol (1.1 mM/0.06 μl)	Rat	mPnO	REM sleep time ↑2× and REM sleep bout frequency ↑2× in 50% of injections; effects blocked by atropine		NSD
	Garzon et al. (35)	Microinjection	Carbachol (0.04–4 μg/0.02 μl)	Cat	vPnO	Induced alternating periods of wakefulness, REM sleep, and REM sleep-like states. REM sleep effects were not dose dependent. REM sleep ↑4–5× (bout duration and frequency ↑2.5×). NREM sleep was suppressed (40–100% reduction). Mixed states: desynchronized EEG, PGO waves, behavioral quiescence with muscle tone (at doses >0.08 μg)		5–10 min
	Boissard et al. (41)	Iontophoretic microinjection	Carbachol (100 mM; 100–200 nA current)	Rat	SubC_A_	At sites where bicuculline/gabazine induced REM sleep carbachol induced a wake-like state with suppressed δ, ⊖, and σ EEG power	↑muscle tone	<5 min
	Pollock and Mistlberger (42)	Microinjection	Neostigmine (8.8 mM/0.05 μl)	Mouse	PnO	Induced wakefulness and suppressed NREM and REM sleep for 3 h post injection; neostigmine induced state characterized by “very low-amplitude” EEG	Suppressed motor activity, occasional circling	Delayed by 3+ h
	Grace et al. (43)	Microdialysis	Carbachol (tissue concentration ~1.8 μg/h)	Rat	PnO/C SubC	Persistent wakefulness/hyperarousal blocked by scopolamine (1 mM)	Asymmetric body flexion, circling, high muscle tone	NA
Local *loss*-of-function	George et al. (13)	Microinjection	Atropine (1 μg) – *blocked the effects of carbachol*	Cat	PnO/C	No “visible effects” reported (*no data shown*)		NA
	Kostowski (15)	Microinjection	Atropine sulfate	Cat	mPnO/C	“Caused no constant behavioral effects” (*no data shown*)		NA
	Gnadt and Pegram (23)	Microinjection	Atropine (0.41 μg/0.1 μl) – *blocked the effects of carbachol*	Rat	PnO/C SubC	No effect on REM sleep time (baseline: 8.75% total recording time vs. atropine: 10% total recording time)		NA
	Imeri et al. (30)	Microinjection	Pirenzepine (M1 antagonist; 1.6 μg/0.1 μl); methoctramine (M2 antagonist; 1–15 μg/0.1 μl); *p*-F-HHSiD (M3 antagonist; 1.6 μg/0.2 μl)	Rat	vPnC	Methoctramine: ↑NREM sleep latency and wake time for three highest doses; REM sleep decreased as a% of total sleep time from ~16 to ~12% on average (across three highest doses); pirenzepine and *p*-F-HHSiD: no effect		↑2–6×Latency relative to first NREM episode
	Bourgin et al. (33)	Microinjection	Atropine (0.1–2 μg/0.1 μl) – *blocked the effects of carbachol*	Rat	PnO/C SubC	No effect on REM sleep time (*no data shown*)		NA
	Marks and Birabil (36)	Microinjection	Atropine (4.9 mM/60 nl) – *blocked the effects of carbachol*	Rat	mPnO	No effect on REM sleep time (*no data shown*)		NA
	Grace et al. (43)	Microdialysis	Scopolamine (M antagonist; 1 mM) – *blocked the effects of* (i)*carbachol and* (ii) selective activation of cholinergic PPT neurons by Urotensin II	Rat	PnO/C SubC	No change in REM sleep time or bout frequency; NREM-to-REM sleep transitions: (i) duration ↑25% and (ii) efficiency ↓ by 30%; The increase in EEG ⊖ power in REM sleep relative to NREM sleep ↓by 25%		NA

**Figure 1:**
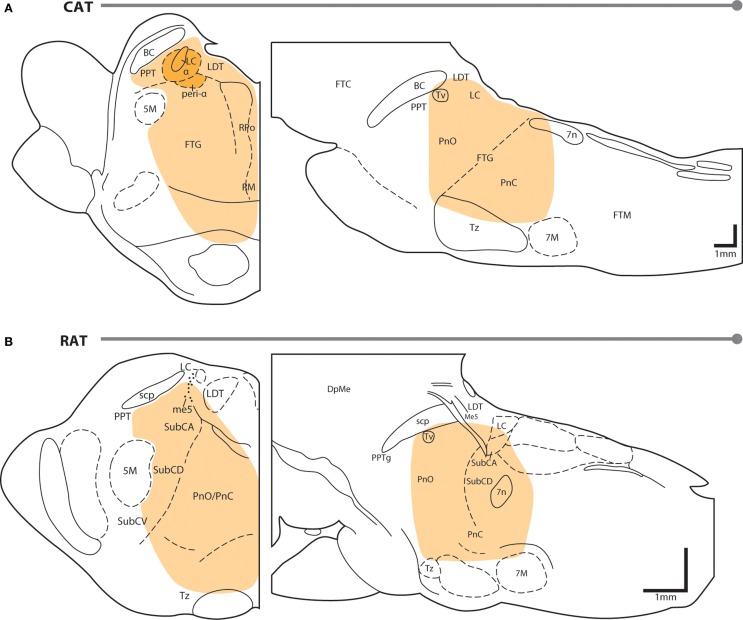
**PTF regions sensitive to cholinomimetic induction of REM sleep**. Coronal (left) and sagittal (right) maps of the cat **(A)** and rat **(B)** pons. The shaded regions are inclusive of all the effective REM sleep inducing injection sites from the studies listed in Table 1. Anatomical abbreviations: 5M, fifth motor nucleus; 7M, seventh motor nucleus; BC, brachium conjunctivum; DpMe, deep mesencephalic reticular nucleus; FTC, central tegmental field; FTG, gigantocellular tegmental field; FTM, magnocellular tegmental field; LC, locus coeruleus; LDT, laterodorsal tegmental nucleus; Me5, mesencephalic 5 nucleus; Peri-LCα, peri-locus coeruleus alpha; PnC, caudal part of the pontine reticular nucleus; PnO, oral part of the pontine reticular nucleus; PPT, pedunculopontine tegmental nucleus; PTF, pontine tegmental field; RM, raphe magnus; RPo, raphe pontis; scp, superior cerebellar peduncle; SubC, subcoeruleus nucleus including alpha (A), dorsal (D), and ventral parts (V); Tv, ventral tegmental nucleus of Gudden; Tz, nucleus of the trapezoid body. Anatomical maps were adapted from Ref. (44, 45).

*A complete chronological listing of pharmacological studies testing the effects on REM sleep of focally delivered cholinomimetic drugs and/or cholinergic receptor antagonists into the pontine tegmental field. In the motor effects column, percentages listed with behaviors indicate the percentage of cases in which that behavior occurred. Anatomical designations correspond to labeled regions enclosed within the shaded areas in Figure 1. Anatomical abbreviations: Peri-LCα, peri-locus coeruleus alpha (cat); PnC, caudal part of the pontine reticular nucleus; PnO, oral part of the pontine reticular nucleus; PTF, pontine tegmental field; SubC, subcoeruleus nucleus including alpha, dorsal and ventral parts (rat). Prefixes: m, medial; d, dorsal; v, ventral*.

##### Neuroanatomy of REM sleep induction sites

Figure 1 shows the region of the PTF in which delivery of cholinomimetic drugs can induce REM sleep in cats (Figure 1A) and rats (Figure 1B). Table 1 provides more specific details about the locations of cholinomimetic drug delivery and the resulting effects on sleep and motor behavior.

Initial studies generated disagreement regarding the location of the pontine site(s) in the cat where cholinergic stimulation generates REM sleep. Studies by Baghdoyan et al. (21, 22) and Hobson et al. (20) reported potent REM sleep inducing effects (40–50 min episodes and four- to ninefold increases in REM sleep time) of cholinergic receptor agonism in the posterior portion the ventral PTF (roughly from the posterior boundary of the laterodorsal tegmental nucleus [LDT; P2 according to Berman (45) to the bisection of the PTF by the facial nerve (P7) and ventral to the level of the trigeminal motor pool]. However, lesioning studies have showed that unlike electrolytic ablation (that destroy neurons and axons of passage) of the ventral PTF, kainic acid (which destroy neurons only) lesions in these regions did not affect REM sleep or its component phenomena (46, 47). These findings suggested that fibers passing through the ventral PTF are important for REM sleep generation rather than the neuronal perikarya. In contrast to posteroventral sites, REM sleep time is greatly reduced or eliminated by chemical lesioning of the anterodorsal pontine tegmentum (ventral to the level of the trigeminal motor pool and anterior to P4) (48–50).

While REM sleep is induced at both anterodorsal and posteroventral PTF sites, the former are associated with inducing REM sleep at the shortest latencies following drug microinjection (i.e., <20 min) (27, 29). Due to drug diffusion following microinjection, longer latencies to REM sleep induction raise the possibility that the site of drug action is at a distance from the delivery site. Gnadt and Pegram (23) showed that a relatively small volume of solution (0.1 μl) containing titrated choline and carbachol spread as much as 1–1.5 mm over the course of an hour. Similarly, Myers and Hoch (51) reported that a 0.5 μl volume of radiolabeled dopamine spread 0.5–1.0 mm after 15 min, while Macklis and Quattrochi (52) found that 0.050 μl of radiolabeled carbachol spread into an area with a 4–5 mm diameter within 1 h of the microinjection. Therefore, longer latencies to REM sleep induction following microinjection into posteroventral PTF sites (injection volume range = 0.25–3 μl) may be the result of the time taken for drug to diffuse to REM sleep generating circuitry located in the anterodorsal PTF. However this interpretation has been challenged by Reinoso-Suarez et al. (32). They found that carbachol microinjections within the dorsal PTF induced persistent waking states punctuated by periodic bouts of muscle atonia, consistent with the findings of Mitler and Dement (38) and van Dongen et al. (17). Only after several hours of such arousal were increases in REM sleep time observed. Moreover, they showed that carbachol microinjections in the ventral portion of the PTF strongly induced REM sleep, with latency to induction being less than 5 min on average. Garzon et al. (35) also found that REM sleep could be induced at a short latency following ventral PTF microinjections of carbachol.

As in cats, REM enhancing sites in the rat extend along an anteroposterior axis from the posterior pole of the ventral tegmental nucleus of Gudden (Tv) to the root of the facial nerve (7n). There is no particular zone within this portion of the rat PTF where REM sleep can be reliably enhanced; responses to cholinergic stimulation range from no effect, to REM sleep enhancement, to arousal and motor impairment (for details on response variety, see the following sections and Table 1). However, despite the lack of reliability, REM sleep enhancing sites do tend to cluster in the subcoeruleus/sublaterodorsal portion of the rat PTF medial to the trigeminal motor pool (34).

##### The differences between cat and rodent studies including wakefulness effects

There are notable differences in the features of cholinergic-induced REM sleep between cats and rodents. Unlike in cats, where REM sleep episodes can be triggered within minutes of drug microinjection, reported latencies to REM sleep induction in rats exceed 30 min (Table 1). As noted previously for cats, episodes of cholinergic-induced REM sleep can last as long as 50 min and that REM sleep time increases to levels 3–9 times normal. By contrast, following PTF cholinergic stimulation in rats, REM sleep time increases to only 1.5–2 times normal levels and extended bouts of REM sleep do not occur (Table 1). Rather, increases in REM sleep time stem from increased bout frequency. Recall that the magnitude of REM sleep effects in cats – “the capacity to overwhelm” – was the primary indicator that cholinergic neurotransmission might play a major role in REM sleep generation. Consequently, the weaker effects of cholinergic stimulation observed in rats have led to alternative hypotheses suggesting that cholinergic neurotransmission in the PTF serves a modulatory, rather than a critical, role in REM sleep generation.

In rodents, the primary effect of cholinergic stimulation is often increased wakefulness rather than increased REM sleep (23, 33, 34, 41). Pollock and Mistlberger (42) reported that microinjection of neostigmine into the PTF of mice induces extended periods of wakefulness characterized by elevated muscle tone, delaying REM sleep onset for up to 3 h. Similarly, Boissard et al. (41) showed that at dorsal PTF sites where GABA_A_ receptor antagonism induced REM sleep and carbachol microinjection induced a waking state characterized by heightened muscle tone. Mastrangelo et al. (31) showed that carbachol microinjections in the rat PTF induced extended periods of wakefulness lasting 20–80 min; thereafter, REM sleep time increased by 40%. In a large mapping study of the rat PTF, Deurveilher et al. (34) showed that carbachol microinjections induced wakefulness as often as REM sleep (13% of cases); 74% of injections produced no effect. Persistent wakefulness following cholinergic stimulation has also been reported in the cat. Baghdoyan et al. (22) found that carbachol microinjections at posterodorsal PTF sites induced wakefulness marked by ataxia, poor hind limb control, and aggressive behaviors. Mitler and Dement (38) showed that carbachol microinjection into the peri-locus coeruleus region of the anterodorsal PTF induced a persistent waking state within 1–10 min of injection that could last upwards of 30 h. In that study, motor atonia did appear but only in 6 of 15 injections after extended periods of wakefulness.

The arousing effects of cholinergic stimulation in the PTF are often accompanied by unusual motor activity. Reporting on the effects of carbachol microinjection into the PTF of the rat, Gnadt and Pegram (23) noted that cholinergic stimulation sometimes produced “ipsilateral contraction of the axial musculature, particularly in the neck and thorax. In its mildest form, this appeared as an ipsiversive circling behavior. In its most severe form, the contracture completely drew the rat into an incapacitating ipsilateral flexion of the body.” Similar results have been reported in cats by van Dongen (19). Reports of increased wakefulness following delivery of cholinomimetics into the PTF are often associated with such motor abnormalities. In the mapping study by Deurveilher et al. (34), induced wakefulness was accompanied by asymmetric body flexion or hyperactive circling in 90% of cases. The association between motor effects and arousal raises the possibility that cholinomimetic-induced arousal is an epiphenomenon of motor disruption. Therefore, particularly in rats, the activation of motor circuitry and the resulting state of arousal may conceal what capacity exists for cholinergic stimulation to induce REM sleep in the PTF. It is important to acknowledge that cholinoceptive REM sleep-related neurons in the pons are intermingled amongst cholinoceptive locomotion-related reticulospinal neurons that provide input to spinal central pattern generators (53). Experimental stimulation of these reticulospinal cells and the mesencephalic locomotor region that drives their activity produces similar patterns of muscle contraction and behavior as those noted above (54).

##### State dissociation

In addition to the study by George et al. (13), several additional studies in cats have reported state dissociation following cholinergic stimulation of the PTF. Cholinergic stimulation produced dissociated REM sleep states at sites scattered throughout the PTF; however, state dissociation was associated most frequently with microinjections in the peri-locus coeruleus region of the anterodorsal PTF (i.e., the same region associated with cholinomimetic-induced motor abnormalities and arousal). Vanni-Mercier et al. (27) reported that carbachol microinjection at points near the peri-locus coeruleus alpha in cats produced several distinct REM sleep-like states. In some cases, cats entered a state very similar to normal REM sleep; however, hippocampal theta activity was more reminiscent of wakefulness being described as medium amplitude and lower frequency (i.e., 3–5 Hz). This state contrasted with periods where carbachol induced a REM sleep-like hippocampal theta rhythm that persisted into non-REM sleep. In other cases, cats entered a state characterized by the occurrence of atonia, EEG desynchronization, ponto-geniculo-occipital waves, and REMs typical of normal REM sleep; however, the animals were otherwise alert, able to track visual stimuli, and orient toward low intensity auditory stimuli. The most common form of state dissociation reported in cats following cholinergic PTF stimulation is persistent motor atonia – occurring in 80% of studies reporting state dissociation.

#### Interpreting Gain-of Function Stimulation Studies

The induction of REM sleep by cholinergic stimulation of the PTF is often regarded as indirect evidence supporting the involvement of endogenous PTF cholinergic neurotransmission in REM sleep generation. Many investigators interpret the results of gain-of-function cholinergic stimulation studies as supporting the same conclusions as loss-of-function experiments, just to a lesser degree. It is our position that this should be considered a misinterpretation of gain-of-function stimulation studies. We submit that, in principle, the results of such cholinergic stimulation do not necessarily support or oppose any measure of endogenous cholinergic involvement in REM sleep generation.

Cholinergic stimulation of the PTF tests whether or not the endogenous cholinergic inputs to this region have a capacity, under the conditions of the experiment, to generate REM sleep or its component parts. In spite of the variable and species-dependent nature of cholinergic REM sleep enhancement, the capacity of endogenous cholinergic inputs to the PTF to generate REM sleep has been established. However, we should not infer the function of cholinergic PTF afferents in REM sleep generation from their demonstrated capacity – our reasoning is as follows (55). The function, if any, of PTF cholinergic afferents in REM sleep generation is a product of the REM sleep control network’s response to their input. This response depends on two factors. The first factor is the initial state of the REM sleep control network at the time of cholinergic input. The second factor is the magnitude (and perhaps the pattern) of the cholinergic input. Therefore, the role of PTF cholinergic afferents in REM sleep generation is ultimately a function of the timing and the intensity of their activation. The refractory period following carbachol-induced REM sleep illustrates how the effect of cholinergic input to the PTF depends on timing and initial conditions. During the refractory period, an additional bolus of carbachol induces a waking state marked by high muscle tone rather than REM sleep (13). Also, optogenetic stimulation of PTF cholinergic afferents in the LDT and pedunculopontine tegmental nucleus (PPT) is only effective at enhancing REM sleep when stimulation occurs in non-REM sleep – waking and REM sleep stimulations have no effect [(56), see “Selective Modulation of PPT and LDT Subpopulations” for details]. Similar results were obtained by Lopez-Rodriguez et al. (40) who showed that direct cholinergic stimulation in the PTF most often induced REM sleep when microinjections were made during non-REM sleep. Subsequent microinjections at the same locations during wakefulness preferentially induced a dissociated state characterized by motor atonia and sensory awareness. The importance of stimulus intensity is illustrated by the fact that, depending on the dose applied, cholinergic input to the PTF can have no effect, induce REM sleep, induce wakefulness, or produce dissociated states. Gain-of-function stimulation experiments simulate the effects of neural activation with intensity and timing that are not necessarily relevant to the physiological case. The effects of cholinomimetic stimulation of the PTF approximate the function of endogenous cholinergic inputs only in the case that those inputs are a major source of inductive REM sleep drive to the PTF. However, *a priori*, it is equally possible that cholinergic inputs are only minor sources of inductive drive relative to other afferents. It is also possible that cholinergic PTF input is non-inductive; the activation of REM sleep generating circuits in the PTF may precede the activation of their cholinergic afferents. In either of these alternative cases (i.e., where cholinergic PTF input is minor or non-inductive), stimulation of the PTF with cholinomimetic drugs could still have a major capacity to induce REM sleep despite endogenous cholinergic input to the PTF being potentially insignificant in the natural initiation of the state. Determining the function of PTF cholinergic inputs in REM sleep generation requires loss-of-function experiments that remove or block this afferent input. This is the only approach that can preserve the sequence of events and the initial conditions leading up to the activation of PTF cholinergic inputs.

##### Case study: optogenetics and the involvement of MCH containing neurons in REM sleep generation

There is an emerging consensus that melanin concentrating hormone (MCH) containing neurons in the hypothalamus play a role in the generation and maintenance of REM sleep. Studies reporting REM sleep enhancement following optogenetic stimulation of MCH neurons are cited in support of this claim. Konadhode et al. (57) have shown that optogenetic stimulation of MCH containing neurons shortens sleep latency and increases time spent in both non-REM and REM sleep at the expense of wakefulness. A similar study by Jego et al. (58) showed that optogenetic activation of MCH neurons during non-REM sleep facilitated non-REM-to-REM sleep transitions, while activation at the onset of REM sleep bouts increased their duration. Likewise, Tsunematsu et al. (59) showed that optogenetic activation of MCH neurons induced transitions into REM sleep and increased REM sleep time at the expense of time spent in non-REM sleep. Many in the field have interpreted these findings as implicating hypothalamic MCH neurons in the mechanism of REM sleep generation (57–62). We submit that while these data demonstrate the capacity of MCH containing neurons to generate and maintain the state of REM sleep, they do not necessarily implicate MCH neurons in this function. More importantly, the role of MCH containing neurons in REM sleep generation ought to be inferred from loss-of-function experiments rather than gain-of-function experiments. However, neither optogenetic silencing (58) nor targeted ablation (59) of MCH neurons has any effect on the generation or maintenance of REM sleep. These loss-of-function silencing experiments demonstrate that MCH neurons do not play a major role in the induction or maintenance of REM sleep, in spite of the REM sleep enhancing effects of MCH neuron stimulation.

### Blockade of cholinergic input to the putative REM sleep generator

Identifying the function of *endogenous* cholinergic inputs to the PTF in REM sleep generation requires the removal or blockade of cholinergic inputs. A small number of studies have examined the effects of antagonizing cholinergic receptors in the PTF. In the seminal study of cholinomimetic-induced REM sleep, George et al. (13) anecdotally reported that, in two cats, atropine microinjections into the PTF had no “visible effects.” Similarly, Gnadt and Pegram (23) microinjected atropine in five rats and reported that it had no effect on REM sleep time (no data provided). Bourgin et al. (33) reported that in three rats, microinjection of atropine alone did not affect REM sleep time at the same sites where carbachol induced REM sleep (no data provided). By contrast, two studies have reported decreases in REM sleep following pharmacological blockade of cholinergic receptors in the pons. Firstly, Shiromani and Fishbein (24) reported significant reductions of REM sleep in rats following pontine infusions of the muscarinic acetylcholine receptor antagonist scopolamine. However, infusions took place continuously over 5 days using a chronically implanted osmotic mini-pump. Over such an extended period of time, the infused scopolamine would have spread far beyond the target site located in the posteromedial PTF. Similar reductions in REM sleep occurred with medullary and ventricular infusions. In a second study, Imeri et al. (30) reported increases in REM sleep latency in rats following pontine microinjections of the muscarinic type-2 receptor antagonist methoctramine. Methoctramine microinjection resulted in a dose-dependent increase in the latency to REM sleep. Total REM sleep time was also reduced following methoctramine microinjections. However, the reduced REM sleep time and the increased REM sleep latencies may have been a secondary effect of arousal, because non-REM sleep time was also reduced while wake time increased, particularly at the highest doses. Taken together, these data neither confirm nor refute the involvement of pontine cholinergic neurotransmission in the generation of REM sleep.

In a recently published study, we aimed to determine the role of endogenous cholinergic input to the PTF in the generation of REM sleep in rats (63).Using bilateral reverse microdialysis of the cholinergic receptor antagonist scopolamine, we showed that blocking cholinergic input to the PTF had no effect on REM sleep time or the frequency of REM sleep episodes. Importantly, the concentration of antagonist used was sufficient to block the effects of carbachol microperfusion (63). In our study, carbachol mainly induced extended episodes of wakefulness marked by abnormal motor behavior [similar to Ref. (17, 23, 34, 38)]. In the event that cholinergic receptor antagonism in the PTF does not block or reduce REM sleep, there is always the possibility that drug delivery was unable to completely block cholinergic input to the PTF. We aimed to minimize this possibility (63). Having previously measured the extent of drug diffusion using reverse microdialysis (43), we would expect microperfused antagonist to permeate the cholinoceptive REM sleep induction zone of the rat (approximately 14 mm^3^) within the recording period. Most importantly, we showed that scopolamine microperfusion in the PTF effectively blocked the REM sleep enhancement produced by selectively activating PTF cholinergic afferents in the PPT (see “Selective Modulation of PPT and LDT Subpopulations” for details) (63). Accordingly, we expect that our microperfusions of scopolamine were sufficient to block the endogenous cholinergic input to REM sleep generating sites in the PTF. Since scopolamine microperfusion into the PTF still had no effect on REM sleep time, we submit that endogenous cholinergic input to the PTF is not involved in the induction of REM sleep (63). Despite not effecting REM sleep time, antagonism of cholinergic inputs to the PTF did increase the duration and failure rate of transitions from non-REM-to-REM sleep (63). Therefore, while cholinergic inputs to the PTF are not required for inducing transitions into REM sleep, they may serve an accessory role in reinforcing transitions after their initiation.

### Cholinergic afferents of the pontine tegmental field

#### Non-Selective Modulation of the PPT and LDT

The claim that cholinergic neurotransmission in the PTF plays a major role in REM sleep generation entails that PTF cholinergic afferents located in the PPT and LDT are principal sources of the inductive drive that initiates REM sleep. PPT and LDT cell groups contain subpopulations of neurons that increase their activity in anticipation of and during REM sleep (i.e., REM sleep-active) (64–66). The activation of cholinergic REM sleep-active PPT and LDT neurons is likely responsible for the increase in endogenous acetylcholine release in the PTF during REM sleep (67). Electrical or pharmacological stimulation of PPT and LDT neurons has been shown to increase REM sleep (66, 68–70), induce acetylcholine release in the PTF (71), and evoke scopolamine-sensitive excitatory postsynaptic potentials in PTF neurons (72). These data further support the claim that cholinergic PTF afferents have a capacity to generate REM sleep.

Determining the function of cholinergic PPT and LDT neurons in REM sleep generation requires loss-of-function experiments. Reductions in REM sleep have been reported following electrolytic or chemical lesioning of the PPT and the LDT in cats, consistent with their causal involvement in REM sleep generation (49, 73). However, in these studies, lesions were not restricted to cholinergic cell areas and are therefore difficult to interpret. Lesions included other important components of the REM sleep generating circuitry, including the deep mesencephalic reticular nucleus (DpMe), the ventrolateral periaqueductal gray (vlPAG), and the anterodorsal PTF including the peri-locus coeruleus region.

The effects of smaller, more selective chemical lesions of the PPT and LDT in rats (50) indicate that neither of these cell groups is needed to generate REM sleep. Lu and colleagues reported that LDT lesions did not affect REM sleep *per se*, but did increase the number of total state transitions per hour. Surprisingly, PPT lesions increased, rather than decreased, REM sleep time. This finding raises the possibility that some PPT subpopulations may function to suppress, rather than promote, REM sleep. An inhibitory influence on REM sleep by PPT neurons is further supported by the finding that pharmacological inactivation of the PPT, by GABA_A_ receptor agonism, increases REM sleep both as a percentage of total recording and total sleep time – i.e., independent of concurrent changes in wakefulness (74–76). By contrast, Petrovic et al. (77) reported no change in REM sleep amounts following bilateral PPT lesions; however, PPT lesions resulted in an increased number non-REM-to-REM sleep and wake-to-REM sleep transitions. Moreover, REM sleep split into two electroencephalographically distinct states (i.e., “sigma coherent” and “theta coherent” REM sleep).

The lack of consistency between PPT/LDT modulating studies notwithstanding, the REM sleep effects of manipulating the PPT or LDT en masse should not be taken to reflect the function of the REM sleep-active cholinergic neurons in these regions. Cholinergic REM sleep-active PPT/LDT neurons are vastly outnumbered by other cell types (78–80). The composition of the LDT and PPT cell groups is highly heterogeneous. While the boundaries of the PPT and LDT are defined by their cholinergic subpopulations, 74–81% of PPT/LDT neurons are GABAergic or glutamatergic (81). Furthermore, immunostaining for c-Fos following REM sleep deprivation and recovery has revealed that only a small proportion (5–15%) of PPT/LDT neurons exhibiting heightened activity in REM sleep are cholinergic (78, 79). Much larger proportions (50–85%) are GABAergic (78, 82, 83). Moreover, Clement et al. (84) have shown that more than 50% of LDT neurons found to be c-Fos positive after REM sleep recovery are glutamatergic.

REM sleep-active neurons in the PPT and LDT can be subdivided into two groups based on their firing rate profile across the sleep–wake cycle (64–66, 80). First, some REM sleep-active neurons exhibit maximal activity in REM sleep and minimal activity in wakefulness (i.e., REM sleep-max active). Within this group, some neurons have firing rates that increase gradually from active wakefulness through non-REM sleep to REM sleep, while other neurons only markedly increase their firing immediately prior to and during REM sleep (80). Second, some REM sleep-active neurons in the PPT and LDT discharge minimally during non-REM sleep. Neurons of this type exhibit increased firing during wakefulness in association with muscle activation, firing rate deceleration in transition to non-REM sleep, and acceleration of firing rate in transition to REM sleep (i.e., REM sleep/wake-max active). Within this group, some neurons discharge maximally in active wakefulness while others discharge maximally in REM sleep (80). In addition to REM sleep-active neurons, some PPT and LDT neurons discharge minimally in REM sleep and maximally during active wakefulness (80). Wake-active subpopulations of the PPT/LDT are hypothesized components of the ascending reticular activating system that maintains wakefulness (85).

Using juxtacellular recording and labeling in the PPT and LDT, Boucetta et al. (80) reported that these groups are neurochemically diverse, consisting of cholinergic, GABAergic, and glutamatergic cell types. Previously, cholinergic neurons in the PPT and LDT were assumed to be both REM sleep-max active and REM sleep/wake-max active. However, Boucetta et al. (80) found that recorded cholinergic neurons in the LDT and PPT were exclusively REM sleep/wake-max active. Since the sample size was small [six neurons; LDT(5) and PPT(1)] further studies are needed to definitively determine whether or not any REM sleep-active cholinergic PPT/LDT neurons are REM sleep-max active. Boucetta et al. (80) showed that the majority (4/6) of REM sleep/wake-max active cholinergic neurons in the LDT and PPT discharged maximally in REM sleep. On average, discharge rates of cholinergic REM sleep/wake-max active LDT and PPT neurons were 63 and 348% greater than those in active and quiet wake, respectively. The existence of PPT and LDT cholinergic neurons that discharge maximally in REM sleep (irrespective of their activity in wakefulness) is also supported by positive immunostaining for c-Fos in cholinergic LDT/PPT neurons following recovery from selective REM sleep deprivation. If REM sleep-active cholinergic neurons in the LDT and PPT were exclusively REM sleep/wake-max active and did not discharge at significantly higher rates in REM sleep as compared to wakefulness, it would be difficult to explain the observations of Maloney et al. (78) and Verret et al. (79) that c-Fos expression increases in rebound versus deprived conditions when the combined time spent in wakefulness and REM sleep remains constant. Taken together, these studies demonstrate that there are distinct subpopulations of neurons defined by unique combinations of neurotransmitter expression and activity profile within the PPT and LDT. These subpopulations could be functionally distinct and therefore the inconsistencies between studies that manipulate these subpopulations en masse could be a reflection of this functional diversity.

#### Selective Modulation of PPT and LDT Subpopulations

Determining the functional importance of individual PPT and LDT subpopulations requires that they be targeted selectively. PPT and LDT cholinergic neurons selectively express receptors for Urotensin II, a vasomodulatory peptide (86). Urotensin II has been shown to selectively excite cholinergic PPT and LDT neurons *in vitro* (87, 88). Local infusion of Urotensin II in the PPT of rats increased the number of REM sleep episodes and the total time spent in REM sleep both as a percentage of total recording and total sleep time (63, 88). Urotensin II microperfusion in the PPT also decreased non-REM-to-REM sleep transition duration and increased non-REM-to-REM sleep transition efficiency (i.e., attempts to transition from non-REM to REM sleep failed less often) (63). The effects of Urotensin II at the PPT were blocked by simultaneous antagonism of cholinergic receptors in the PTF (63). Consistent with the effects of selective pharmacological activation of PPT and LDT cholinergic neurons, selective optogenetic activation of PPT and LDT cholinergic neurons increased the frequency of REM sleep episodes as well as time spent in REM sleep, at the expense of time spent in non-REM sleep (56). Optogenetic stimulation increased the probability of transitioning into REM sleep only when stimulation occurred in non-REM sleep. REM sleep was unaffected by stimulations occurring in wakefulness or REM sleep (56), for commentary see Ref. (55, 89). Taken together, these gain-of-function interventions confirm that, free of the potentially confounding effects of modulating intermingled non-cholinergic neurons, cholinergic PPT and LDT neurons have the capacity to enhance non-REM-to-REM sleep transitioning and induce REM sleep. These data also show that, at least in the case of PPT cholinergic neurons, this capacity for REM sleep enhancement is exerted through cholinoceptive PTF neurons. However, we submit that these studies do not necessitate that cholinergic LDT and PPT neurons are involved in the induction of REM sleep. We do not know what would be the effect, if any, of selectively inhibiting cholinergic PPT and LDT subpopulations – e.g., optogenetic inactivation or selective neurotoxic lesioning with Urotensin II conjugated to diphtheria toxin (90). However, the effects of cholinergic antagonism at the PTF (see Blockade of Cholinergic Input to the Putative REM Sleep Generator for details) would suggest that PPT and LDT cholinergic inputs to the PTF increase the efficiency of non-REM-to-REM sleep transitioning, but are otherwise not needed for the occurrence of REM sleep (63).

REM sleep-max active PPT and LDT neurons can be selectively inhibited by the 5-HT_1A_ agonist 8-hydroxy-2-(di-n-propylamino) (8-OH-DPAT), leaving the activity of REM sleep/wake-max unaffected (65). Surprisingly, selective inhibition of the REM sleep-max active subpopulation of the PPT increased REM sleep, as a percentage of total recording and total sleep time (91). The increase in REM sleep time stemmed from an increase in the frequency of REM sleep episodes particularly during periods of low REM sleep drive/propensity (91) quantified electroencephalographically using a modified algorithm developed by Benington and Heller (92) and Benington et al. (93). Recall that, based on c-Fos immunostaining following REM sleep deprivation and recovery, PPT REM sleep-active neurons are predominately GABAergic and glutamatergic. It is possible that, PPT subpopulations exert opposing yet complementary influences of REM sleep. Non-cholinergic REM sleep-max active PPT neurons may function to raise the drive threshold for REM sleep induction in order to limit REM sleep episodes to periods of high propensity. Following the onset of a transition into REM sleep, cholinergic PPT neurons may be recruited to reinforce switching and increase the probability of transition success (63). We hypothesize that the combined action of these PPT subpopulations may function to reduce the sensitivity of the REM sleep switch to noise.

## The REM Sleep Switch

### The reciprocal interaction hypothesis

**Figure 2:**
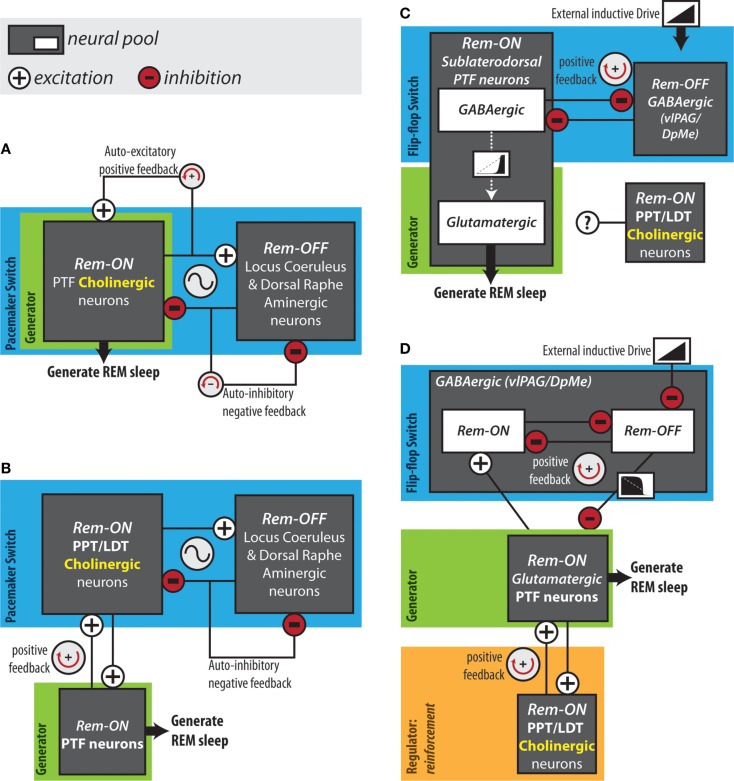
**Hypothesized REM sleep control circuits**. **(A)** The original reciprocal interaction hypothesis. **(B)** Modified reciprocal interaction hypothesis. **(C)** Flip-flop circuit proposed by Lu et al. (50). **(D)** Version of flip-flop circuit proposed by Sapin et al. (83), modified by Grace et al. (63). Anatomical abbreviations: DpMe, deep mesencephalic reticular nucleus; LDT, laterodorsal tegmental nucleus; PPT, pedunculopontine tegmental nucleus; PTF, pontine tegmental field; vlPAG, ventrolateral periaqueductal gray.

Mapping the neuroanatomical network responsible for REM sleep generation, although necessary, is insufficient for an understanding of the network dynamics that actually give rise to cycling into and out of REM sleep. In 1975, Hobson and McCarley proposed the reciprocal interaction model: a structural/mathematical hypothesis meant to provide an explanation for the cyclical generation of REM sleep. This model posited that a reciprocal interaction between REM sleep-inactive cell groups and REM sleep-active cell groups form a pacemaker circuit that drives oscillations between sleep stages (94) (Figure 2A). Aminergic neurons in the locus coeruleus and dorsal raphe were hypothesized as the REM sleep-inactive cells. REM sleep-active neurons in the PTF innervating the LC were presumed to be cholinergic and excitatory (95). The model proposed that during wakefulness “cholinergic” PTF neurons would be inhibited by activated aminergic neurons. At non-REM sleep onset, waning aminergic neuron activity would disinhibit “cholinergic” PTF neurons. At a critical point, the combination of this disinhibition and auto-excitation within the “cholinergic” cell group would enable the exponential rise in PTF neuron activity that triggers the onset of REM sleep (96). During REM sleep, “cholinergic” PTF neurons would excite aminergic neurons resulting in their own inactivation and the termination of the REM sleep episode.

The reciprocal interaction model was proposed before cholinergic neurons could be immunohistochemically identified. The discovery that PTF neurons are predominately non-cholinergic forced a revision of the model (97) (Figure 2B). REM sleep-active cholinergic neurons in the PPT and LDT were included as the main REM sleep promoting group (96, 98). In the revised model, PPT and LDT cholinergic neurons triggered REM sleep by exciting REM sleep-related non-cholinergic neurons in the PTF. In this version of the structural model, a mutually excitatory connection between the PTF and the PPT/LDT was responsible for the exponential rise in PTF neuron activity at REM sleep onset (95, 99). This pacemaker circuit was modeled mathematically using equations of the Lotka–Volterra type derived from population models of predator–prey interactions (94). The simple Lotka–Volterra model formed the basis for the more robust limit cycle model, which incorporated circadian modulation (100). The time course of neuronal activity in the REM sleep-active and REM sleep-inactive cell groups predicted by the mathematical models, coincide with the actual long-term recordings of REM sleep-active and REM sleep-inactive neurons. However, models of this type have been criticized on the basis that they rely on the tuning of unconstrained parameters to achieve a particular ultradian period. Several lines of evidence argue against the reciprocal interaction model as being a workable explanation for REM sleep generation. Most notably, loss-of-function interventions targeting the structural components of the model – the PPT, LDT, LC, DRN, and cholinergic neurotransmission in the PRF – have little or no effects on the occurrence of REM sleep (50, 63, 74–76, 91).

### The flip-flop hypothesis

More recently, it has been suggested that mutual inhibition between REM sleep-active and REM sleep-inactive GABAergic cell groups creates a flip-flop switch that is critical for REM sleep generation (50, 83). Unlike a pacemaker circuit (e.g., reciprocal interaction model), which is intrinsically capable of generating an ultradian rhythm, a flip-flop switch is triggered by an input signal external to the circuit. In other words, pacemaker network models assume that the periodicity of the sleep cycle is an intrinsic property of the circuit’s topology, whereas a flip-flop mechanism assumes that cycling between sleep stages occurs as a response to external demands. A flip-flop switch can convert a graded input signal, which changes slowly over time, into a two-state output that reduces time spent in intermediate states (i.e., bistability) (101). In comparison with a pacemaker model, a flip-flop mechanism can better account for (i) the occurrence of a “rebound” after REM sleep deprivation (102–105) and (ii) the positive correlation between the length of a REM sleep bout and the length of the preceding non-REM bout (this correlation would likely be negative in the case of a pacemaker mechanism) (92).

In 2006, Lu and colleagues proposed that REM sleep is generated by a mutually inhibitory interaction between REM sleep-active GABAergic neurons in the sublaterodorsal region of the PTF and REM sleep-inactive GABAergic neurons in the vlPAG and the adjacent DpMe (Figure 2C). In support of this arrangement, retrograde and anterograde tracing experiments showed that the sublaterodorsal PTF and vlPAG/DpMe neuronal pools mutually innervate one another (50). Moreover, Boissard et al. (106) have shown that DpMe and vlPAG projections to the sublaterodorsal PTF are GABAergic; Lu et al. (50) have demonstrated that half of the sublaterodorsal pontine tegmental cells retrogradely labeled from the DpMe and vlPAG contain GAD67 mRNA.

In contrast to sublaterodorsal PTF inactivation, which suppresses REM sleep, vlPAG and DpMe inactivation potently increases REM sleep time, increasing the frequency and length of REM sleep episodes (50, 83, 107, 108). While these effects are consistent with those that one would expect in the case that the sublaterodorsal PTF and vlPAG/DpMe neuronal pools form a mutually inhibitory flip-flop switch, it has been shown that REM sleep-active neurons in the sublaterodorsal PTF are predominately glutamatergic rather than GABAergic (84). It is therefore unlikely that REM sleep-active sublaterodorsal PTF neurons form a mutually inhibitory flip-flop switch together with vlPAG/DpMe neurons. Sapin et al. (83) alternatively proposed that a mutually inhibitory flip-flop switch might be formed between GABAergic REM sleep-inactive vlPAG/DpMe neurons and the large number of GABAergic REM sleep-active neurons also located in the vlPAG/DpMe (Figure 2D). In either case, activation of REM sleep-related PTF neurons and initiation of REM sleep would result from the withdrawal of GABAergic inhibition by REM sleep-inactive vlPAG/DpMe neurons. Consistent with such an inductive mechanism, GABA_A_ receptor antagonism in the PTF terminates non-REM sleep and induces persistent REM sleep (41, 109–111).

Flip-flop hypotheses of REM sleep generation remain speculative; however, a recent modeling study has tested the theoretical validity of flip-flop switch involvement in the generation of the sleep cycle. Dunmyre et al. (112) constructed a physiological model based on coupled flip-flop oscillators for the control of transitioning between wakefulness, non-REM and REM sleep. With the inclusion of separate non-REM and REM sleep “homeostatic” drives, the modeled circuit was able to faithfully reproduce experimentally measured rodent sleep architecture under normal and sleep-deprived conditions. Importantly, unlike previous flip-flop models of behavioral state transitioning (113, 114), this study does not incorporate intrinsic oscillatory capability into the neuronal groups. Therefore, this study provides theoretical validation that “homeostatically” regulated interlocking flip-flop oscillators are sufficient to explain behavioral state transitioning.

### A hypothesis for the structural basis of the cholinergic effects on non-REM-to-REM sleep transitioning

It was previously noted that strong evidence has confirmed the capacity of cholinergic neurotransmission in the PTF to induce REM sleep; however, blockade of this cholinergic neurotransmission does not prevent REM sleep. Instead, we found evidence that cholinergic input to the PTF reinforces transitions making them quicker and less likely to fail. We also noted that the reciprocal interaction model posits that a mutually excitatory (i.e., positive feedback) interaction between cholinergic LDT/PPT neurons and the PTF causes the exponential rise in PTF neuron activity in advance of REM sleep onset. Consistent with the existence of such a mutually excitatory positive feedback loop, (i) neuroanatomical evidence shows that the PTF and LDT/PPT cell groups innervate one another (115–117) and (ii) unilateral carbachol stimulation of the PTF causes endogenous acetylcholine release in the contralateral pontine tegmentum (118), possibly due to positive feedback onto PPT/LDT neurons from cholinoceptive PTF neurons.

Given that REM sleep time and the frequency of REM sleep bouts are both unaffected by cholinergic inputs to the PTF, it is unlikely that this cholinergic input provides inductive drive to trigger the activation of REM sleep-related PTF neurons. In other words, in the sequence of events giving rise to REM sleep, it is unlikely that PPT/LDT activation precedes PTF activation. The available evidence is best explained in the case where the activation of PPT/LDT cholinergic neurons is gated by activity in the PTF, and where the PPT/LDT provides cholinergic positive feedback to PTF neurons. In this case, cholinergic receptor antagonism could have little or no effect on REM sleep quantity despite there being a major capacity for exogenous cholinergic stimulation to initiate REM sleep or its component parts (see Case Study: Optogenetics and the Involvement of MCH Containing Neurons in REM Sleep Generation for further discussion of this point). Consistent with this hypothesis, the increase in firing rates of REM sleep-active GABAergic and glutamatergic neurons in the pontomesencephalic tegmentum that occurs during the non-REM to REM sleep transition period precedes that of cholinergic LDT/PPT neurons (80).

Using computer modeling, we have shown that a positive feedback loop producing this sequence (i.e., PTF → PPT/LDT → PTF) can account for the transition reinforcement produced by PTF cholinergic neurotransmission *in vivo* (63) (Figure 2D). Using the flip-flop circuit proposed by Sapin et al. (83), we showed that varying “cholinergic” positive feedback to the “PTF” did not delay transition onset and therefore did not affect the activation threshold of the “PTF,” likely a major determinant of REM sleep quantity *in vivo*. Reducing “cholinergic” positive feedback to the “PTF” did lengthen transitions into REM sleep as observed *in vivo* (63). Under conditions of reduced positive feedback, peak firing rates of “PTF” neurons were also suppressed. Reduced firing of PTF neurons could underlie our observation that the normal increase in EEG theta power occurring in REM sleep was suppressed during cholinergic receptor blockade in the PTF (63). The PTF contains glutamatergic neurons projecting to the medial septum and lesioning these cells eliminates theta oscillations during REM sleep (50). Lastly, simulations showed that “cholinergic” positive feedback dampened potentially destabilizing surges in flip-flop switch REM-off neuron activity that could underlie transition failure *in vivo* (63).

Our hypothesis that a positive feedback mechanism underlies the function of PTF cholinergic afferents in REM sleep generation does not necessarily require that the upstream switching element be a flip-flop switch. However, in the case that the main switching element is a positive feedback flip-flop circuit, our suggestion of an accessory cholinergic positive feedback loop takes on special significance. Note that while only a single positive feedback loop is required to create bistability in a signaling pathway, nested feedback loops are nevertheless common in biology. For instance, the polarization of cell growth in yeast (119), mammalian calcium signaling (120), and the maturation of xenopus oocytes (121) all utilize interlocking positive feedback loops. Using multiple interlocked feedback loops produces more reliable switches that are rapidly inducible and resistant to extrinsic noise (122).

## Conclusion

(1) The capacity of PTF cholinergic afferents to generate REM sleep has been firmly established by gain-of-function experiments. (2) The function of endogenous PTF cholinergic input in REM sleep generation cannot be determined by gain-of-function experiments; rather, loss-of-function studies are required. (3) Loss-of-function studies show that endogenous cholinergic input to the PTF is not required for REM sleep generation. (4) Cholinergic input to the PTF serves an accessory role in REM sleep generation: reinforcing non-REM-to-REM sleep transitions making them quicker and less likely to fail.

## Conflict of Interest Statement

The authors declare that the research was conducted in the absence of any commercial or financial relationships that could be construed as a potential conflict of interest.
